# Label-Free Quantitative Proteomic Analysis of Harmless and Pathogenic Strains of Infectious Microalgae, *Prototheca* spp.

**DOI:** 10.3390/ijms18010059

**Published:** 2016-12-29

**Authors:** Jayaseelan Murugaiyan, Murat Eravci, Christoph Weise, Uwe Roesler

**Affiliations:** 1 Institute of Animal Hygiene and Environmental Health, Centre for Infectious Medicine, Freie Universität Berlin, Robert-von-Ostertag-Str. 7-13, 14163 Berlin, Germany; Uwe.Roesler@fu-berlin.de; 2 Institute for Chemistry and Biochemistry, Freie Universität Berlin, Thielallee 63, 14195 Berlin, Germany; Murat.eravci@gmx.de (M.E.); chris.weise@biochemie.fu-berlin.de (C.W.)

**Keywords:** *Prototheca*, protothecosis, proteomics, comparative proteomic analysis, label free quantitative analysis, LTQ Orbitrap Velos mass spectrometer, MaxQuant, Perseus

## Abstract

Microalgae of the genus *Prototheca* (*P.*) spp are associated with rare algal infections of invertebrates termed protothecosis. Among the seven generally accepted species, *P. zopfii* genotype 2 (GT2) is associated with a severe form of bovine mastitis while *P. blaschkeae* causes the mild and sub-clinical form of mastitis. The reason behind the infectious nature of *P. zopfii* GT2, while genotype 1 (GT1) remains non-infectious, is not known. Therefore, in the present study we investigated the protein expression level difference between the genotypes of *P. zopfii* and *P. blaschkeae.* Cells were cultured to the mid-exponential phase, harvested, and processed for LC-MS analysis. Peptide data was acquired on an LTQ Orbitrap Velos, raw spectra were quantitatively analyzed with MaxQuant software and matching with the reference database of *Chlorella variabilis* and *Auxenochlorella protothecoides* resulted in the identification of 226 proteins. Comparison of an environmental strain with infectious strains resulted in the identification of 51 differentially expressed proteins related to carbohydrate metabolism, energy production and protein translation. The expression level of Hsp70 proteins and their role in the infectious process is worth further investigation. All mass spectrometry data are available via ProteomeXchange with identifier PXD005305.

## 1. Introduction

Ubiquitously present achlorophyllous microalgae of the genus *Prototheca* (*P*) spp. (family Chlorellaceae) are associated with rare but severe infections in animals and humans termed protothecosis. With the recent reclassification of a strain isolated from a human patient, the genus *Prototheca* currently includes seven generally accepted species, among which *P. blaschkeae*, *P. cutis*, *P. miyajii*, *P. wickerhamii* and *P. zopfii* have been reported to be associated with infections [1,2,3,4,5]. Furthermore, *P. cutis*, *P. miyajii* and *P. zopfii*, *P. wickerhamii* have been reported as associated with human protothecosis, while *P. blaschkeae*, *P. wickerhamii* and *P*. *zopfii* were reported to be the most common species involved in animal protothecosis including the dreadful bovine mastitis [4,5,6].

*P. zopfii* has been divided into two genotypes, the non-pathogenic *P. zopfii* genotype 1 (GT1) and the infection-associated genotype 2 (GT2). However, the reason behind the pathogenic nature of *P. zopfii* GT2 remains to be elucidated. *P. zopfii* genotypes display a clear difference in biochemical, serological, genetic, and proteomic analysis [7,8]. It was demonstrated that GT2 alone was able to induce clinical symptoms upon experimental infection of cows [9], and experimental infection with primary bovine mammary epithelial cells indicated the stronger adhesion of GT2 than GT1 [10]. While *P. zopfii* GT2 is the agent considered to be associated with severe infection, because it is the most frequently encountered organism during protothecal bovine mastitis, *P. blaschkeae* (earlier designated as biotype 3 of *P. zopfii*) represents a mildly infectious agent associated with subclinical infection and isolated only sporadically [2,4,9,11,12,13,14]. Despite the association of *P. zopfii* GT2 and *P. blaschkeae* with bovine mastitis, their difference in pathogenicity, their reservoir in the cow shed and its surroundings and the *Prototheca* genome sequence is not known [2,15]. The strains of the same species—despite their genomic similarity—often display a varying virulence pattern; therefore, the quantification of protein expression among the genotypes of *P*. *zopfii* is important to understand their mechanisms of infection. Earlier proteomic studies indicated that the adaptation of GT2 to an intracellular life occurs via adjusting its metabolism and signal transduction [16,17]. Proteomic analysis of *P. zopfii* genotypes and a comparison with *P. blaschkeae* might help us to better understand the pathogenic nature of *Prototheca* spp. In the present study, label-free quantitative proteomics was applied to detect differences in the protein expression between non-pathogenic *P. zopfii* GT1 and those species associated with severe and subclinical bovine mastitis, *P. zopfii* GT2 and *P. blaschkeae*.

## 2. Results and Discussion

Microalgae of genus *Prototheca* have been associated with severe infections in vertebrates. The taxonomy of *Prototheca* is continuously expanding, and currently seven species have been generally accepted as *Prototheca* species. *P. zopfii* is the most virulent *Prototheca* species, associated with human protothecosis and bovine mastitis. Quantitative proteomic studies using two-dimensional difference gel electrophoresis (DIGE)/MALDI-TOF MS analysis and application of the liquid chromatography tandem mass spectrometry (LC-MS/MS)-based isobaric tag for relative and absolute quantitation (iTRAQ) technique indicated that the infectious microalgae favored an adaptation to an intracellular lifestyle [16,17]. Two-dimensional electrophoresis-based serological proteome analysis (SERPA) using sera collected from experimentally infected rabbits and naturally infected dogs revealed that *Prototheca* also possess antigenic proteins well known from other eukaryotic pathogens [18,19]. Despite the clear description of *P. zopfii* genotypes and these proteomic analyses, both the mechanism of infection and the reservoir of infectious strains remain to be elucidated. In the present study, a label-free quantitative proteomic technique was applied to investigate the protein expression in three different strains, a non-pathogenic strain (*P. zopfii* GT1), the most frequently isolated strain from the infected cases (*P. zopfii* GT2), and a mildly infective strain (*P. blaschkeae*).

### 2.1. Label-Free Quantitative Proteomics

Label-free quantitative proteomic analysis demands highly reproducible LC-MS/MS platforms and provides a higher number of differentially expressed proteins compared to other quantitative approaches due to its higher sequence coverage [20]. This method also allows the identification of proteins with extreme molecular weight, pI and hydrophobic properties, which are otherwise difficult to detect using conventional gel-based approaches. In the present study, a reference database of the closely related species *Chlorella variabilis* and *Auxenochlorella protothecoides* was chosen, as *Prototheca* represents an “orphan species” whose genome sequence has not yet been sequenced.

A total of 245 proteins were identified, among which 226 proteins (42% and 58% belonging to *Chlorella* and *Auxenochlorella*, respectively) were valid after removal of known contaminants. Another criterion for successful protein identification was the presence of the signal in at least four out of six samples in every group, where each group represents one genotype or species. The entire dataset of protein identification is given as a supplementary table (Table S1). The identified proteins covered a molecular weight range from 7 to 255 kDa. In addition, to demonstrate the presence of extremely hydrophobic proteins, the GRAVY (grand average of hydropathy) value was calculated by adding the hydropathy value for each residue and dividing it by the length of the sequence. While the hydropathy values of the majority of the proteins ranged from −2 to +2, more positive values indicated an extremely hydrophobic nature of the proteins. Among the identified proteins, 18 proteins possessed a theoretical pI >11, 16 proteins a calculated molecular weight exceeding >120 kDa, and four proteins a GRAVY score >2.0. Despite the capability to include such extreme proteins, the results described here represent only a tiny fraction of the total database entries, which highlights the difficulties that one might face when working with those organisms whose genome is not sequenced and underscores the importance of obtaining the fully sequenced genome.

**Figure 1:**
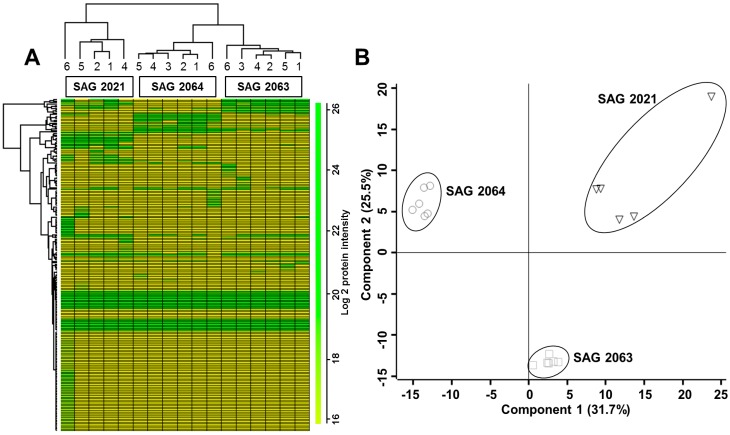
(**A**) Heat map shows the unsupervised hierarchical clustering of proteins from SAG 2021, SAG 2063 and SAG 2064 and all six replicates are clustered together. The log_10_ value of the MS signal intensity is shown; (**B**) Principal component analysis of logarithmized values without z-scoring indicating the strains used for the analysis.

As shown in Figure 1, hierarchical clustering analysis of the data sets using elucidation as the distance and the average as the linkage for 226 quantified proteins, displayed three major clusters representing genotypes of *P. zopfii* and *P. blaschkeae*. Among the six samples of SAG 2021, one sample was lost during the sample processing while all five samples included in the analysis appear to cluster distinctly, but the replicates still stayed within the same cluster. Further principal component analysis displayed three different groups, and among SAG 2021, sample 6 remained different from the other samples but was still grouped at a distance.

### 2.2. Differentially Expressed Proteins

With statistical analysis using Perseus software and the application of Student’s *t*-test, a *p*-value less than 0.05% and 1% false discovery rate (FDR) resulted in the identification of 51 differentially expressed proteins (Table 1), among which several proteins were consistent with the earlier 2D-DIGE and SERPA studies [16,18,19]. A two-way Student-*t* test was performed to identify proteins that were differentially expressed between (a) SAG 2021 and SAG 2063; (b) SAG 2064 and SAG 2063; and (c) SAG 2021 and SAG 2064.

The comparison of SAG 2063, a non-pathogenic, environmentally isolated strain, with SAG 2021, the strain most frequently isolated from bovine mastitis, indicated 28 differentially expressed proteins, among which 20 and eight proteins were upregulated and downregulated, respectively, in the pathogen strain SAG 2021. Further, the comparison of the non-pathogenic SAG 2063 strain with the mildly infectious agent *P. blaschkeae* SAG 2064 resulted in the identification of 29 (14 upregulated and 15 downregulated) differentially expressed proteins. These two comparisons display differences between a severely and a mildly infectious strain and a non-infectious strain. The third comparison represents the difference between the mild (SAG 2064) and severe (SAG 2021) infection-associated strains, in which 35 (18 upregulated and 17 downregulated) proteins were identified as differentially expressed in SAG 2064.

**Figure 2:**
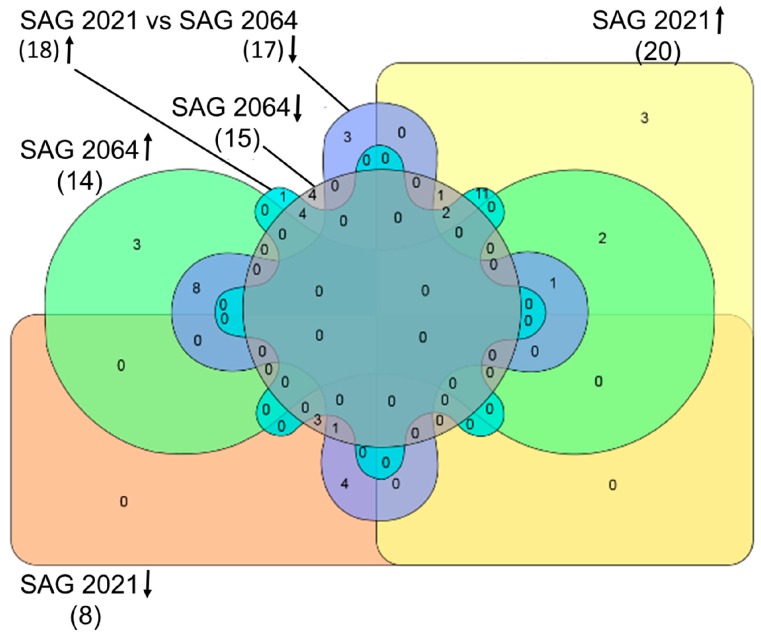
Venn diagram created using InteractiVenn, a web-based tool to compare the accession numbers of proteins identified. Total number of proteins is listed within the parenthesis, up- and down-arrows indicate up- and down-regulated proteins, respectively. Colour codes: yellow-SAG 2021 up-regulated, orange-SAG 2021 down-regulated, green-SAG 2064 up-regulated, purple-SAG 2064 down-regulated, blue-SAG 2021 up-regulated and grey SAG 2064 down-regulated.

InteractiVenn, a web-based tool used to display overlaps between data sets (Figure 2), revealed that three proteins in SAG 2021 (heat shock protein 70, 40S ribosomal protein S27 and an uncharacterized protein) and SAG 2064 (histone H4 and two uncharacterized proteins) were uniquely upregulated. SAG 2021 did not show any unique protein downregulated when compared to non-infectious strains; however, the mildly infectious strain displayed downregulation of four proteins (ubiquitin-60S ribosomal protein L40-2, glucose-6-phosphate isomerase, nucleoside diphosphate kinase 1, elongation factor Tu). On the other hand, one uncharacterized protein and three proteins (enolase, chaperone protein ClpB1 and GTP-binding nuclear protein) appeared to be upregulated and downregulated, respectively, between the mildly infectious SAG 2064 and the severely infectious SAG 2021 strains.

Among seven differentially expressed proteins, three (V-type H^+^ ATPase, fructose-bisphosphate aldolase and an uncharacterized protein) and four proteins (a member of heat shock protein 70, citrate synthase, acetyl-coenzyme A synthetase and uncharacterized protein) were respectively upregulated and down-regulated in both SAG 2021 and SAG 2064. Among these proteins, fructose-bisphosphate aldolase and a member of heat shock protein 70 appeared to be upregulated in SAG 2021 when compared to SAG 2063.

### 2.3. Functional Annotation

**Figure 3:**
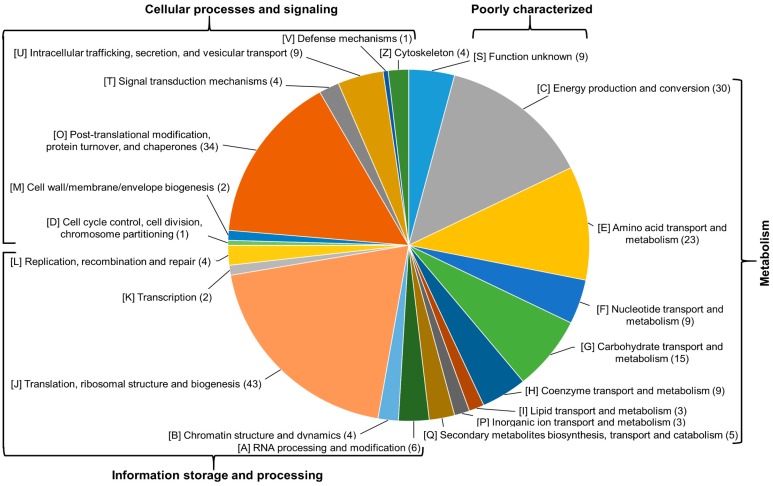
Distribution of proteins based on their Cluster of Orthologous Group (COG) annotation. The number in the brackets at each category represents the actual number of proteins.

The eggNOG classification revealed that the identified proteins belong to four functional groups: cellular processes and signaling (D, M, O, T, U, V and Z), information storage and processing (A, B, J, K and L), metabolism (C, E, F, G, H, I, P and Q), and poorly characterized (R) (Figure 3). An assignment to a Cluster of Orthologous Group (COG) was not possible for four proteins (E1ZQB3, E1Z2U9, E1Z360 and A0A087SQC7) for which the query sequence was too large, and three proteins (E1Z2F4, A0A087SPK7 and A0A087SHV8) returned with no possible COG.

The comparison between the environmental and the pathogenic strains revealed that the major differences lie in (G) carbohydrate transport and metabolism, (C) energy production and conversion, (J) translation, ribosomal structure and biogenesis and (O) post-translational modification, protein turnover, and chaperones. Even though this result is in accordance with earlier reports [16,17], one should be cautious with the interpretation based on functional classification: since the majority of the identified proteins were connected to these functions, the majority of the differentially expressed proteins also tend to be in the same categories.

Interestingly, the well-known antigen of other eukaryotic pathogens, glyceraldehyde-3-phosphate dehydrogenase (A0A087SBQ6), did not show any difference between the *P. zopfii* genotypes, but was significantly upregulated in *P. blaschkeae*. This trend was also observed in an earlier flow-cytometric analysis [3].

Heat-shock protein 70, identified as up-regulated in infectious strains, is worth further investigation. Proteins of the HSP70 family have been described as immuno-dominant antigens that elicit an immune response to infectious diseases caused by bacteria, protozoa, fungi and nematodes [21]. In the present study, both heat-inducible (A0A087SND2, E1Z7R4, E1ZQV2 and E1ZE03) and constitutively expressed members (A0A087SJ70: heat shock cognate 70 kDa protein) of heat shock protein 70 were identified. Three among the five identified members of the HSP70 family were differentially expressed among *P. zopfii* genotype 2 and *P. blaschkeae* while the other two, heat shock protein 70B (E1ZE03) and heat shock cognate 70 kDa protein (A0A087SJ70)h were not differentially expressed but were predicted by the VaxiJen v2.0 server as potential antigens.

Despite the significance of these results, they represent only a tiny fraction of the database entries. Further research mimicking the infection environment and sequence information is needed to better understand *Prototheca* infection–associated pathways.

## 3. Materials and Methods

### 3.1. Cell Culture and Protein Extraction

Three strains, *P. zopfii* genotype 1 (SAG 2063^T^), *P. zopfii* genotype 2 (SAG 2021^T^) and *P. blaschkeae* (SAG 2064^T^) [2] were chosen from the culture collection at the Institute of Animal Hygiene and Environmental Health, Freie Universität Berlin, Germany. These strains are also available at the Culture Collection of Algae at the University of Göttingen (SAG), Göttingen, Germany. Additional information on these strains can be found elsewhere [2]. Each strain was cultured six times independently in Sabouraud dextrose liquid medium (Oxoid, Wesel, Germany) for about 20–24 h until the OD_600_ reached 0.6 (mid-logarithmic growth phase). The cells were harvested by centrifugation at 11,290× *g* for 5 min, the supernatant was removed and the pellet was washed three times by cycles of resuspension and centrifugation in 1.0 mL of phosphate buffered saline. The resultant cell pellet was inactivated by adding 300 µL of distilled water and 900 µL of ethanol, mixed and centrifuged at 11,290× *g* for 2 min, the supernatant discarded and the pellet was allowed to air dry completely. The final pellet was reconstituted with 250 µL of 20 mM HEPES (pH 7.4) and subjected to sonication on ice for 1 min (cycle, 1.0; amplitude, 100%) with a sonicator (UP100H; Hielscher Ultrasound Technology, Teltow, Germany). The suspension was centrifuged at 11,290× *g* for 5 min at 4 °C. The clear supernatant was collected, protein concentration was measured using modified Bradford’s method (Biorad, Germany) and stored at −20 °C until further analysis.

### 3.2. In Solution Trypsin Digestion

The protein extract containing 10 µg of proteins was subjected to acetone precipitation by adding a fivefold volume of ice-cold acetone, incubated at −20 °C for 10 min, centrifuged at 1700× *g* for 10 min at 0 °C, supernatant discarded and the resulting precipitate was allowed to air-dry. The precipitate was then reconstituted with 20 µL of denaturation buffer (6 M urea/2 M thiourea in 10 mM HEPES, pH 8.0) and reduced for 30 min at room temperature with 0.2 µL of 10 mM dithiothreitol in 50 mM of ammonium bicarbonate (ABC). The alkylation was carried out by adding 0.4 µL of 55 mM iodacemtamide in ABC and incubation for 30 min at RT. Subsequently 0.4 µL of LysC (Wako, Neuss, Germany, Germany) solution (0.5 µg/µL in ABC) was added and incubated overnight. The urea concentration was decreased by adding 75 µL of ABC, 0.4 µL of 0.5 µg/µL trypsin in 50 mM ABC was added and incubated overnight. The trypsin digestion was stopped by adding 100 µL of 5% acetonitrile/3% trifluoroacetic acid.

### 3.3. Liquid Chromatography–Electrospray Ionization–Tandem Mass Spectrometry (LC–ESI–MS/MS)

The resultant peptides of trypsin digestion were desalted by solid phase extraction using C_18_ Empore disc cartridges (Supelco/Sigma-Aldrich, Taufkirchen, Germany) [22]. Desalted peptide mixtures were separated by reverse-phase chromatography using the Dionex Ultimate 3000 nanoLC (Dionex/Thermo Fisher Scientific, Idstein, Germany) on in-house manufactured 25 cm fritless silica micro-columns with an inner diameter of 100 μm. Columns were packed with the ReproSil-Pur C18-AQ 3 µm resin (Dr. Maisch GmbH, Entringen, Germany). Peptides were separated on a 5%–60% acetonitrile gradient (90 min) with 0.1% formic acid at a flow rate of 350 nL/min. The eluted peptides were ionized online by electrospray ionization and transferred into an LTQ Orbitrap Velos mass spectrometer (Thermo Fisher Scientific, Bremen, Germany) which was operated in the positive mode to measure full scan MS spectra (from *m*/*z* 300–1700 in the Orbitrap analyzer at resolution *R* = 60,000) followed by isolation and fragmentation of the twenty most intense ions (in the LTQ part) by collision-induced dissociation.

### 3.4. Protein Identification

The raw MS/MS spectra search were processed using a freely available software suit, MaxQuant (version. 1.3.0.5). Initial maximum precursor and fragment mass deviations were set to 7 ppm and 0.5 Da, respectively. Variable modification (methionine oxidation and N-terminal acetylation) and fixed modification (cysteine carbamidomethylation) were set for the search and trypsin with a maximum of two missed cleavages was chosen for searching. The minimum peptide length was set to 7 amino acids and the false discovery rate (FDR) for peptide and protein identification was set to 0.01.

Proteins from the *Chlorella variabilis* (Green alga) (Proteome ID: UP000008141, protein count: 9831, version May 2016) and *Auxenochlorella protothecoides* (Green microalga) (Proteome ID: UP000028924: protein count: 7001, version May 2016) proteome were identified after the protein reference proteomes were downloaded from the UniProt Knowledgebase and imported into the MaxQuant-associated Andromeda search engine [23]. Matching against the protein sequence database was carried out after improving the precursor ion mass accuracy using the time- and mass-dependent recalibration option of the software. The false-discovery rate was controlled at various levels by using a target-decoy search strategy, which integrates multiple peptide parameters such as length, charge, number of modifications and the identification score into a single quality that acts as the statistical evidence on the quality of each single peptide spectrum match [23,24]. The frequently observed laboratory contaminants were removed and the protein identification was considered valid only when at least one unique or “razor” peptide was present. Following protein identification, the intensity for each identified protein was calculated using peptide signal intensities. As described in much detail in [25], in the MaxLFQ label-free quantification method a retention time alignment and identification transfer protocol (“match-between-runs” feature in MaxQuant) is applied and a novel algorithm is used to extract the maximum possible quantification information.

### 3.5. Differentially Expressed Proteins and Statistical Analysis

The freely available software Perseus (version 1.4.1.3) (Available online: http://141.61.102.17/perseus_doku/doku.php?id=start) was used to compare the peak intensities across the whole set of measurements to obtain quantitative data for all of the peptides in the sample. The LFQ intensities of proteins from the MaxQuant analysis were imported and transformed to logarithmic scale with base two. The missing values were replaced (imputated) with the value of the lowest intensity. The protein quantification and calculation of statistical significance was carried out using two-way Student-*t* test and error correction (*p* value < 0.05) using the method of Benjamini*–*Hochberg. For further visualization, heat-map and Principle component analysis (PCA) were performed. All those proteins that showed a fold-change of at least 1.5 and satisfied *p* > 0.05 were considered differentially expressed.

The theoretical molecular weight and iso-electric point (pI) of the identified proteins were obtained from the UniProt database and freely available online software (Available online: http://www.gravy-calculator.de) was used to calculate the grand average of hydropathy (GRAVY) value. The FASTA files of protein sequences were analyzed using http://eggnogdb.embl.de (assessed on September 2016) to achieve the functional annotation of the identified proteins in terms of clusters of orthologous group (COG) [26]. The freely available VaxiJen v2.0 (Available online: http://www.jenner.ac.uk/VaxiJen) server was used to compute the antigenic propensities of proteins solely based on their physicochemical properties. VaxiJen prediction was independent of sequence alignment and in the present study “parasite” model with a threshold of 0.5 was chosen for the prediction [27].

For better understanding, the differentially expressed proteins were identified in comparison among genotypes of *P. zopfii* and with *P. blaschkeae*. The up- and down-regulated protein datasets were included in InteractiVenn, a web-based software, to create a Venn diagram and to identify proteomic level differences among these three strains [28].

The mass spectrometry proteomics data have been deposited to the ProteomeXchange Consortium [29] via the PRIDE partner repository with the dataset identifier PXD005305.

## 4. Conclusions

In the present study, a label-free quantitative proteomic technique was applied to detect protein expression level differences between the genotypes of *P. zopfii* and *P. blaschkeae*. The reference database of the related algal species *Chlorella variabilis* and *Auxenochlorella protothecoides*, downloaded from the Uniprot database, was utilized for protein identification through cross-matching. The number of identified proteins turned out to be negligible compared to the vast number of protein entries in the database; however, the identified proteins were in line with earlier proteomic studies. Further proteomic analysis will be needed, using a *Prototheca* sequence–specific database and culturing of the strains at varying temperatures, to mimic the protein expression within the animal system as well as in the environment to better understand the infection-associated pathways. The role of members of the HSP70 protein family in the infection process is worth further investigation.

## Figures and Tables

**Table 1:** List of the differentially expressed proteins: S. No: serial number as number assigned α-betically to entry. Acc. No. is the accession number and proteins names are as listed in the Uniprot Knowledgebase database. Regulation: only those proteins which are significantly regulated and after filtering 1% false discovery rate (FDR). (+) indicates upregulated and (−) indicates downregulated.

S. No	Acc. No.	Protein Names	Regulation		
			SAG2021 (GT2) vs. SAG2063 (GT1)	SAG2064 (GT3) vs. SAG2063 (GT1)	SAG2021 (GT2) vs. SAG2064 (GT3)
1	A0A087SJM7	40S ribosomal protein S10	(+)		(+)
2	E1ZGA3	40S ribosomal protein S27	(+)		
3	E1ZQY4	40S ribosomal protein S5	(+)		(+)
4	A0A087SBU8	5-methyltetrahydropteroyltriglutamate-homocysteine methyltransferase	(+)		(+)
5	A0A087SNV1	60S ribosomal protein L12-1	(+)		(+)
6	A0A087SKG6	60S ribosomal protein L6	(+)		(+)
7	A0A087SN43	6-phosphogluconate dehydrogenase, decarboxylating (EC 1.1.1.44)	(+)		(+)
8	A0A087SI38	Acetyl-coenzyme A synthetase		(+)	(−)
9	E1ZLA8	Acetyl-coenzyme A synthetase (EC 6.2.1.1)	(−)	(−)	
10	A0A087SS91	Aconitate hydratase, mitochondrial (Aconitase) (EC 4.2.1.3)	(+)	(−)	
11	A0A087SSM0	Actin	(−)		(−)
12	A0A087SF19	Adenosylhomocysteinase (EC 3.3.1.1)		(−)	(+)
13	A0A087SJV3	Aldehyde dehydrogenase family 2 member B4, mitochondrial	(+)	(−)	(+)
14	A0A087SJX6	Argininosuccinate synthase	(+)		(+)
15	A0A087SBN0	ATP synthase subunit beta (EC 3.6.3.14) (EC 4.2.1.24)		(+)	(−)
16	A0A087SPA9	Carbamoyl-phosphate synthase large chain	(+)		(+)
17	A0A087SAK4	Chaperone protein ClpB1			(−)
18	A0A087SQR3	Chaperonin CPN60, mitochondrial		(+)	(−)
19	A0A087SCT6	Citrate synthase	(−)	(−)	
20	A0A087SFG0	Cysteine synthase, chloroplastic/chromoplastic	(−)		(−)
21	A0A087SK74	Elongation factor 1-α		(−)	(+)
22	A0A087SE71	Elongation factor Tu		(−)	
23	A0A087S9L8	Enolase			(−)
24	A0A087SHS8	Eukaryotic initiation factor 4A-10	(+)		(+)
25	A0A087SP16	FK506-binding protein 1	(−)		(−)
26	E1ZTB0	Fructose-bisphosphate aldolase (EC 4.1.2.13)	(+)	(+)	(−)
27	A0A087SG29	Glucose-6-phosphate isomerase (EC 5.3.1.9)		(−)	
28	E1ZFZ5	Glutamate dehydrogenase	(+)		(+)
29	A0A087SBQ6	Glyceraldehyde-3-phosphate dehydrogenase, cytosolic		(+)	(−)
30	A0A087SI84	GTP-binding nuclear protein			(−)
31	A0A087SND2	Heat shock 70 kDa protein, mitochondrial		(+)	(−)
32	E1Z7R4	Heat shock protein 70	(+)		
33	E1ZQV2	Heat shock protein 70	(−)	(−)	(−)
34	A0A087S9W3	Histone H4		(+)	
35	A0A087SSF2	Nucleoside diphosphate kinase 1		(−)	
36	A0A087SQ68	Phosphate carrier protein, mitochondrial	(+)		(+)
37	A0A087ST26	Phosphoglycerate kinase (EC 2.7.2.3)		(+)	(−)
38	E1Z5R3	Putative uncharacterized protein		(−)	(+)
39	E1ZCI5	Putative uncharacterized protein	(+)	(+)	
40	E1ZD41	Putative uncharacterized protein			(+)
41	E1ZG37	Putative uncharacterized protein	(+)	(−)	(+)
42	E1ZL24	Putative uncharacterized protein	(−)	(−)	
43	E1ZMD2	Putative uncharacterized protein		(+)	
44	E1ZRV3	Putative uncharacterized protein		(+)	
45	E1ZSM6	Putative uncharacterized protein	(+)		
46	A0A087SNN6	Stress-induced-phosphoprotein 1		(+)	(−)
47	A0A087SIY9	Succinyl-CoA ligase (ADP-forming) subunit α-1, mitochondrial		(+)	(−)
48	E1ZJM1	Tubulin β chain		(−)	(+)
49	E1ZK88	Ubiquitin	(−)		(−)
50	A0A087SL21	Ubiquitin-60S ribosomal protein L40-2		(−)	
51	E1ZT42	V-type H+ ATPase subunit A	(+)	(+)	

## Supplementary Materials

Supplementary materials can be found at www.mdpi.com/1422-0067/18/1/59/s1.
